# Gated Recurrent Unit Network for Psychological Stress Classification Using Electrocardiograms from Wearable Devices

**DOI:** 10.3390/s22228664

**Published:** 2022-11-10

**Authors:** Jun Zhong, Yongfeng Liu, Xiankai Cheng, Liming Cai, Weidong Cui, Dong Hai

**Affiliations:** 1 School of Biomedical Engineering (Suzhou), University of Science and Technology of China, Hefei 230026, China; 2 Suzhou Institute of Biomedical Engineering and Technology, Chinese Academy of Sciences, Suzhou 215163, China

**Keywords:** psychological stress, electrocardiogram, heart rate variability, gated recurrent unit, VR high-altitude experiment, wearable devices

## Abstract

In recent years, research on human psychological stress using wearable devices has gradually attracted attention. However, the physical and psychological differences among individuals and the high cost of data collection are the main challenges for further research on this problem. In this work, our aim is to build a model to detect subjects’ psychological stress in different states through electrocardiogram (ECG) signals. Therefore, we design a VR high-altitude experiment to induce psychological stress for the subject to obtain the ECG signal dataset. In the experiment, participants wear smart ECG T-shirts with embedded sensors to complete different tasks so as to record their ECG signals synchronously. Considering the temporal continuity of individual psychological stress, a deep, gated recurrent unit (GRU) neural network is developed to capture the mapping relationship between subjects’ ECG signals and stress in different states through heart rate variability features at different moments, so as to build a neural network model from the ECG signal to psychological stress detection. The experimental results show that compared with all comparison methods, our method has the best classification performance on the four stress states of resting, VR scene adaptation, VR task and recovery, and it can be a remote stress monitoring solution for some special industries.

## 1. Introduction

When one’s ability cannot match the requirements of the external environment, psychological stress will appear, such as too difficult a work task or too heavy a financial burden [1]. In fact, we all live under stress, and moderate stress can keep us competitive. However, chronically living under high stress will increase the risk of physical and psychological disease [2], including severe cardiac arrhythmias, high blood pressure, stroke, gastric ulcers, cancer and depression [3,4]. If people could get their stress situation in a low-cost and convenient way and manage it appropriately, it would not only reduce people’s risk of disease but also improve people’s efficiency, creativity and security at work, especially for special industry practitioners, such as military personnel, pilots, firefighters and high-speed rail drivers. Therefore, it is of great value and of social significance to develop a non-invasive stress estimation system to monitor people’s stress changes in their daily work.

At present, the main basis for psychological stress assessment includes social media information and physiological signals. For the former, it is easy to understand that people’s psychological stress can be roughly estimated by multimodal fusion and analysis of information such as texts, images, and videos posted on social media, and many methods have been proposed in this research direction [5,6]. Further, it is easier for people to obtain social media data than physiological signals. However, the accuracy of its stress assessment depends on how active users are on social media, and it seems difficult to make accurate stress assessments for users who are less active on social media. In addition, because of psychological defense mechanisms, people are likely to deliberately disguise their real stress situations in their behavioral performance. Compared with social media data, physiological signals can provide more objective and reliable information for psychological stress assessment [7]. Physiological signals used for stress assessment mainly include electroencephalogram (EEG), electrodermal activity (EDA), photoplethysmographic (PPG) and electrocardiogram (ECG). Although EEG can provide useful information for psychological stress analysis with high temporal resolution [8], the wearing process of its collection equipment is cumbersome and requires the help of professionals. Moreover, the EEG signal is easily disturbed by movements during the collection process. Therefore, EEG is not suitable for daily monitoring of human psychological stress. Compared with EEG, the acquisition equipment for PPG and EDA is portable, and the acquisition process is simple. However, after being interfered with by body movements, the signal is prone to a large degree of distortion, which will increase the difficulty of subsequent feature extraction and analysis. ECG offers advantages over PPG in terms of stability and reliability and is by far the most widely used cardiac monitoring method in healthcare. In recent years, with the development of wearable devices, many wearable ECG devices with both comfort and anti-interference have been developed, including vests, bracelets and chest belts [9,10,11]. The development of these non-invasive ECG devices is the basis for research on the daily monitoring of people’s psychological stress. Wearable physiological parameter monitoring equipment has also been widely used in the field of human action recognition, which has some implications for our research [12,13,14,15].

Compared with psychological stress detection methods based on scales or social media data, the use of wearable devices to collect ECG signals and detect psychological stress obviously has more advantages in real-time and flexibility of usage scenarios. In practical applications, we can use this solution to monitor the psychological stress state of police, firefighters, pilots and other special industry workers during the execution of tasks in real-time and even give real-time psychological intervention at the right time to relieve their anxiety. This not only can improve their work efficiency but also probably play an important role in keeping them safe. In addition, this solution can also be used in the recruitment and selection of workers in special industries.

When changes in the external environment make people feel tense or anxious, it will also cause a physiological response in the body. At this time, the parasympathetic branch of the human autonomic nervous system (ANS) is temporarily suppressed, and the sympathetic branch is activated, which causes a rapid increase in heart rate, cardiac contractility, blood pressure and respiration, and promotes hormone release [16]. It puts the body in a state of hyperactivity to cope with the upcoming challenge. The changes in the ANS associated with psychological stress can be obtained by recording the ECG signal of the subject. Specifically, these ANS changes can be obtained by HRV (Heart Rate Variability) analysis [17].

In this field, previous studies have mostly used classical machine learning methods to detect psychological stress through HRV features, namely, binary classification of stressed and unstressed [18,19,20,21,22,23,24]. First of all, such a binary classification is not completely consistent with people’s stress experience in real life, and it is more and more necessary to study the evaluation methods of human stress in different states. Secondly, deep learning methods have achieved good results in many fields, such as image recognition, natural language processing and signal processing, so the powerful representation ability of deep learning methods can achieve good results in the multi-classification of psychological stress is a problem worth studying. Furthermore, the generation of psychological stress is not instantaneous, and whether its temporal features can be used to improve the accuracy of psychological stress classification is also an interesting problem.

To this end, in this paper, we introduce an ECG dataset collected under four stress states and propose to introduce the concept of time series into psychological stress assessment in order to improve the classification accuracy. Specifically, by constructing a continuous HRV time series, we use a multi-layer GRU network to extract multi-level features related to psychological stress and finally obtain the results through a classification network composed of multi-layer perceptrons. The contributions of this paper are mainly in two aspects. The first is that we propose the concept of time series in the classification of psychological stress states and introduce a recurrent neural network into the classification of psychological stress to obtain the representation of psychological stress in continuous HRV sequences to improve the classification accuracy. The second is that we conducted a psychological stress data collection experiment with 80 participants, designed and developed a stress-induced VR high-altitude scene and collected ECG signals from the subjects during four stress states, including resting, VR scene adaptation, VR high-altitude task and recovery. The purpose is to construct a dataset that can be used to study the mapping relationship between ECG signals and psychological stress in various states. After data cleaning and elimination, this dataset finally contains the ECG data and corresponding status labels of 63 subjects.

## 2. Related Works

Compared with the subjective scales used in the past, psychological stress assessment based on physiological signals has advantages in objectivity and reliability. In the field of psychological stress or emotion estimation based on physiological signals, many methods have been proposed, and some scholars have put forward their insights and analysis on the relationship between physiological signals and psychological stress.

Classical machine learning methods are widely used to classify psychological stress or emotions. Ref. [18] uses Principal Component Analysis (PCA) to verify the HRV time domain, frequency domain and statistical features and then classify two emotions and five emotions by Support Vector Machines. Ref. [21] selects robust HRV features through the mRMR method, reduces the differences in physiological parameters between individuals through baseline data to improve the classification accuracy, and finally, classifies psychological stress in relaxation and task states through a variety of machine learning methods. In the study of driver stress detection, ref. [25] proposes the use of an enhanced random forest classifier to monitor driver stress by combining ECG waveform features and HRV features. Ref. [23] tries to use various machine learning algorithms, including KNN and multi-layer perceptron (MLP), to classify the psychology stress level using the HRV obtained from the ECG signal, and achieved good classification results through the MLP method. Ref. [26] uses the multi-scale analysis method to evaluate the stress of pilots flying at night by fusing the area of the heart rate curve and constructing the functional relationship between the stress intensity and the training frequency, which effectively improved the effect of high-altitude training. Some researchers use genetic algorithm, artificial bee colony algorithm and improved particle optimization algorithm to optimize multi-kernel support vector machine, which improves the accuracy of stress detection [22].

At the same time, there are also studies that use biochemical indicators as a reference in the experiment and apply a variety of physiological signals to the detection of psychological stress. Ref. [27] proves that some indicators of HRV (e.g., HF, LF) have a strong correlation with some features of the EEG signal (e.g., LAPFpl) for stress estimation by analyzing the linear correlation between the HRV features of the ECG signal and the EEG signal features. Based on the above study, the authors propose that combining EEG with HRV can improve the accuracy of psychological stress detection. Ref. [19] develops a wearable multiphysiological parameter system to measure human stress and collect salivary cortisol as a reference. Specifically, the MAST (Maastricht Acute Stress Test) experiment is used to induce the generation of psychological stress, PCA and statistical methods are used to select and reduce the dimensionality of the features extracted from the recorded ECG, EDA and EEG signals, and finally, the SVM is used to classify psychological stress during the experimental period and the relaxation period. In addition, the experimental results in the paper show that salivary cortisol levels are highly correlated with HRV features. Some researchers also propose the detection of rest and task states of the human body by combining HRV features and PPG waveform features. A wrapping method based on ensemble learning is designed for feature selection, and a decision tree-based bagging model is developed for final state classification [20]. In [28], the salivary amylase and salivary cortisol concentrations are used to label the stress of subjects in TSST experiments into three levels, and the fuzzy ARTMAP method and voting integration method optimized by genetic algorithms have been used to establish a predictive model from subject HRV to psychological stress level, and good accuracy rates have been obtained.

In recent years, the use of deep learning methods to classify psychological stress has gradually emerged. Ref. [24] uses a one-dimensional convolutional neural network to extract the complex features of the RR intervals, thereby building an end-to-end neural network model to detect stress states through ECG signals. The RR interval is the time interval between two adjacent R waves in the ECG signal; that is, the time interval between two heartbeats. Ref. [29] proposes the use of a Gabor wavelet transform and discrete Fourier transform to convert the ECG signal into pictures in the time-frequency domain and frequency domain, respectively, and fuse the original signal, time-frequency domain and frequency domain information through a convolutional neural network to classify five levels of stress. Ref. [30] designs a deep convolutional neural network with a transformer mechanism to detect psychological stress using the location information of R-waves in ECG signals and achieves good performance through the fine-tuned network. Ref. [31] proposes the concept of real-time monitoring of psychological stress, and a convolutional neural network is used for the real-time recognition of acute cognitive stress from ECG signals with a 10-s window, which reduces the detection error rate compared to traditional methods. In previous studies, we used a multi-layer GRU network for the heartbeat classification of ballistocardiogram (BCG) signals and a bidirectional LSTM method for end-to-end heart rate estimation of BCG signals in a regression way, which achieved the best results compared to previous algorithms [32,33]. The successful application of a recurrent neural network in heartbeat detection also inspires and helps us in this work.

## 3. Materials

In this section, the wearable ECG signal collection device, VR scene, the process of the experiment and the dataset will be introduced in detail.

### 3.1. Smart ECG T-Shirt

**Figure 1:**
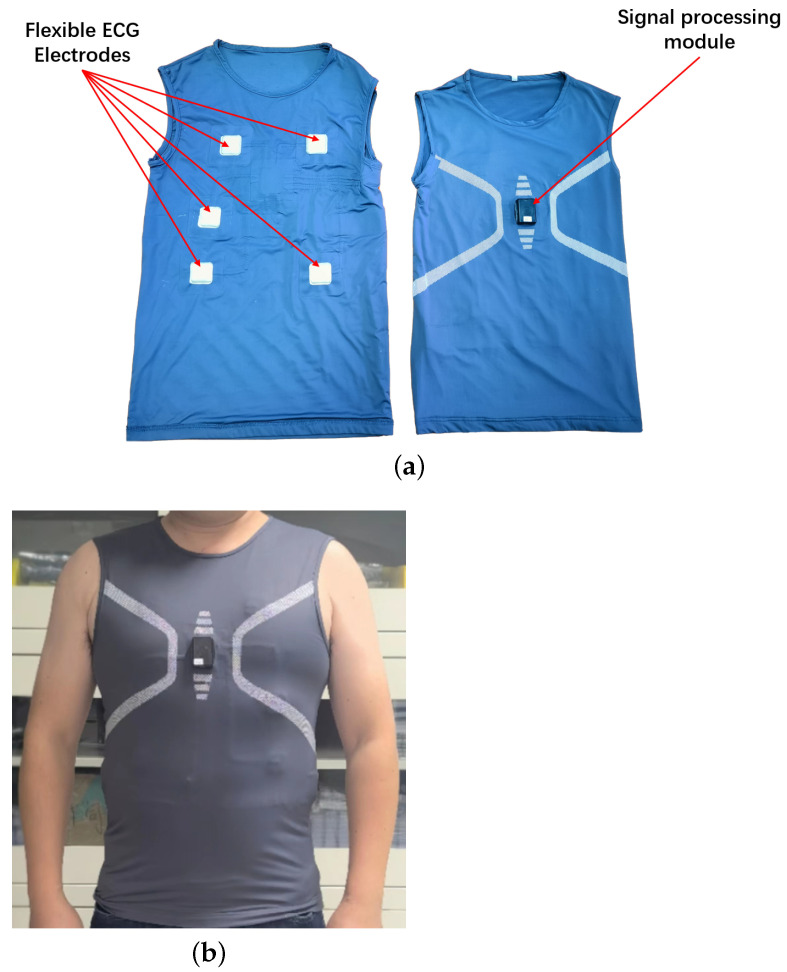
The smart ECG T-shirt. (**a**) The main modules of the smart ECG T-shirt. (**b**) A subject wearing the smart ECG T-shirt.

**Figure 2:**
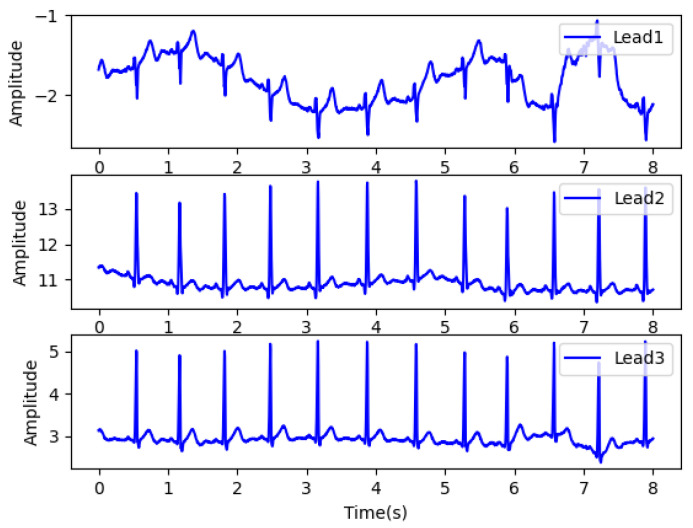
Three-lead ECG signal collected by smart ECG T-shirt.

Figure 1 is the smart ECG T-shirt designed and developed in our laboratory, which can simultaneously record various human physiological signals such as ECG, respiration and electrodermal activity [34]. The left and right of Figure 1a show the front lining and the front of the smart ECG T-shirt, respectively. In the experiment, it is used to record the ECG signals of subjects under different stress states. The sensor system of the smart ECG T-shirt is shown in the left half of Figure 1a, which consists of five flexible electrodes. The right half of Figure 1a shows the signal processing module of the smart ECG T-shirt, which can collect and store three lead ECG signals at a sampling rate of 250 Hz and provide power for the entire system through the built-in lithium battery. Figure 1b shows a subject wearing the smart ECG T-shirt. Figure 2 shows the three-lead ECG signal collected by this device. Each prominently raised peak in Figure 2 represents a heartbeat, and the heartbeat location is consistent across each lead. The recording of three-lead ECG signals can guarantee the signal quality of ECG to a large extent and improve the tolerance of our ECG acquisition equipment to motion or noise interference.

### 3.2. VR Scenarios and Tasks

**Figure 3:**
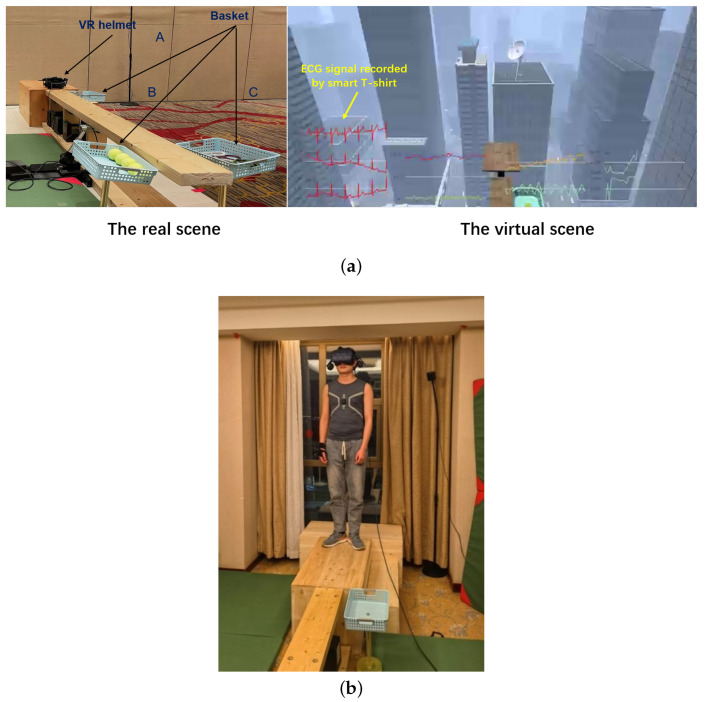
VR experiment. (**a**) Experimental scene and VR scene. (**b**) A subject performing the VR task.

Figure 3 visualizes the VR experiment. The left of Figure 3a shows the experimental scene, and the right shows the VR scene seen by the subjects (in which the curves of various physiological parameters will not be seen). The positions and sizes of key objects in the experimental scene are consistent with the VR scene. During the experiment, the subjects need to wear a VR helmet to enter the virtual high-altitude scene and complete the following three tasks on the board in this scene, as shown in Figure 3b. These three tasks are described in detail as follows:

***Task 1***: Go to the end of the board to pick up the tennis ball from basket B and put it in basket A. Basket A and basket B are shown in the left of Figure 3a.

***Task 2***: Go back to the end of the board to pick up the prop snake from basket C and put it in basket A. Basket A and basket C are shown in the left of Figure 3a.

***Task 3***: Go to the end of the board and jump to the square board shown in the right of Figure 3a.

### 3.3. Experimental Procedure and Dataset

The experiment consisted of four phases: resting, VR scene adaptation, VR task and recovery. The ECG signals were recorded synchronously in each phase of the experiment. Each of these phases is described in detail as follows:

***Phase 1—Resting (5 min)***: Sit calmly in a chair. This phase lasts 5 min.

***Phase 2—VR scene adaptation (2 min)***: Wear VR equipment to enter the VR high-altitude scene, and adapt to the scene. This phase lasts 2 min.

***Phase 3—VR task***: Complete the tennis ball and prop snake transport and jump to the board in the VR high-altitude scene. The duration of this phase depends on how fast the subject is performing the task.

***Phase 4—Recovery (5 min)***: After the VR task, stay calm and sit back in the chair. This phase lasts 5 min.

The above four experimental phases correspond to the four stress states of the subjects. This experiment collects the ECG signals of 63 healthy male subjects with an average age of 17.89 ± 0.45. Considering the adaptability of the subjects and the possible duration of each stress state, we select the subjects’ ECG data in the first 70 s in the VR scene adaptation and recovery states and the subjects’ data in the last 70 s in the resting and VR task states as the stress state classification dataset. Different stress states are the stress classification labels of the corresponding ECG signals so that we can obtain a stress state classification dataset consisting of the ECG data of 63 subjects and four labels. By summarizing the intuitive feelings of each subject in the experiment, we found that the stress level during resting is the lowest, the stress during the VR task is the highest, and the stress during recovery is greater than that in the VR scene adaptation.

## 4. Proposed Method

**Figure 4:**
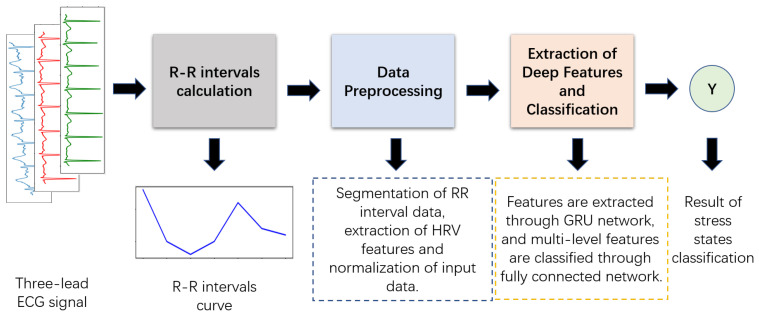
The overall process of the deep classification method based on the GRU network proposed in this paper.

**Figure 5:**
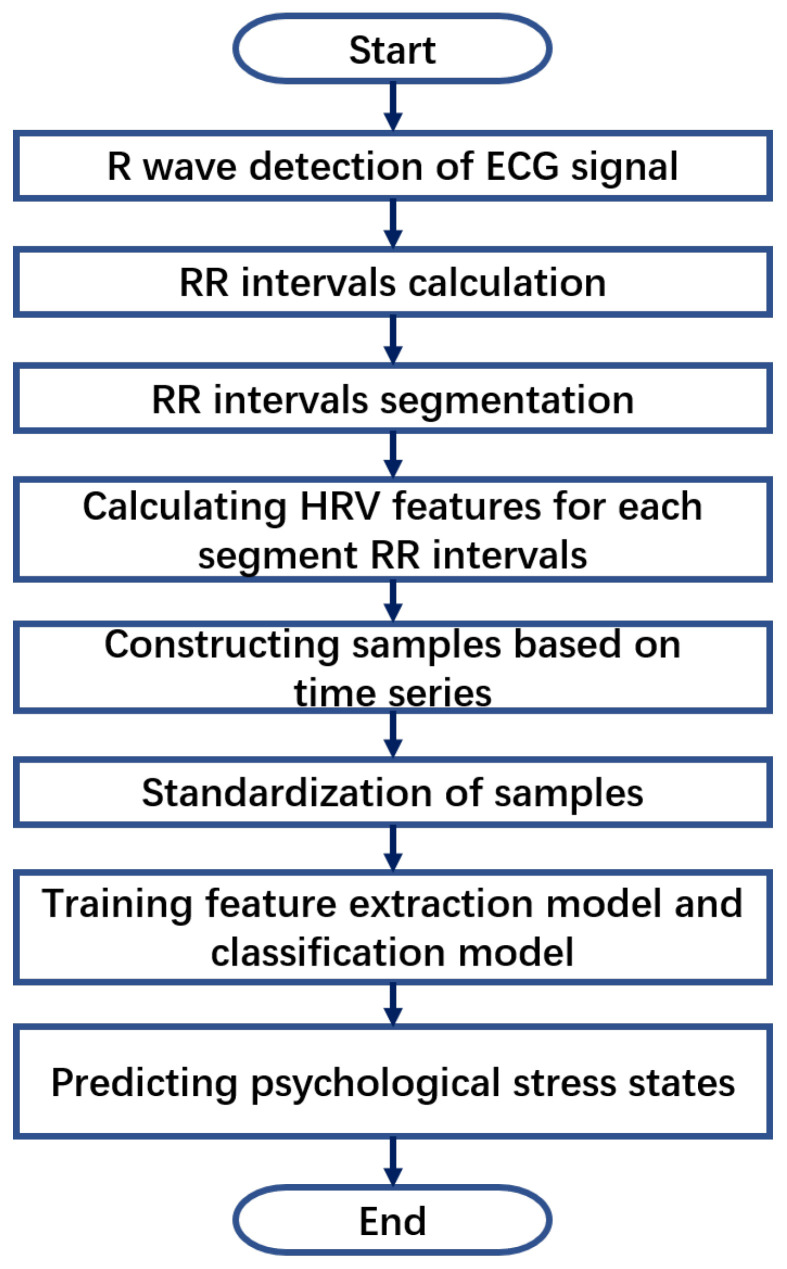
The flow chart of the proposed method.

The purpose of our proposed deep GRU network is to perform the classification of four stress states through ECG signals collected by smart ECG T-shirts. In the HRV feature extraction stage, first, the R waves are detected, and the RR intervals are extracted from the ECG signal, and then the RR interval data under each stress state is divided into fixed-length data segments and arranged in time series. Finally, multiple HRV features, including time domain, frequency domain and entropy information, are extracted from the RR interval data of each segment. In the data preprocessing stage, considering the different physical meanings and numerical dimensions of each HRV feature, the time series relationship between the same HRV feature and the requirements of the input and optimization of the recurrent neural network, we standardize each feature of a single sample at each time by calculating the maximum and minimum values of each HRV feature of all samples at each moment. In the stage of deep feature extraction and stress classification, we design a deep feature extraction model composed of a multi-layer GRU network and a classifier composed of a multi-layer, fully connected network for time series feature extraction and classification of four stress states. The overall process of the proposed deep classification method is shown in Figure 4, and each of these processes is elaborated as follows. Figure 5 is a flow chart of the proposed method, which can make the process of each stage in Figure 4 easier to understand.

### 4.1. HRV Feature Extraction

In this section, we first use a fourth-order Butterworth bandpass filter with a cutoff frequency of 10–35 Hz to filter out high-frequency noise and low-frequency perturbations generated by limb movements in each subject’s ECG signal. Then, the locations of the R waves in the ECG signal are detected, and the RR intervals are calculated for each subject in each stress state. Finally, after the analysis and comparison of previous studies on the classification of psychological stress [18,19,20,21], we select seven HRV features to represent the information of ECG signals, namely, mRR and SDNN containing their time domain information, HFn, LFn and LF/HF containing RR intervals frequency domain information, ApEn containing their entropy information and their nonlinear feature SD1/SD2. The description of each feature is shown in Table 1.

### 4.2. Data Preprocessing

**Figure 6:**
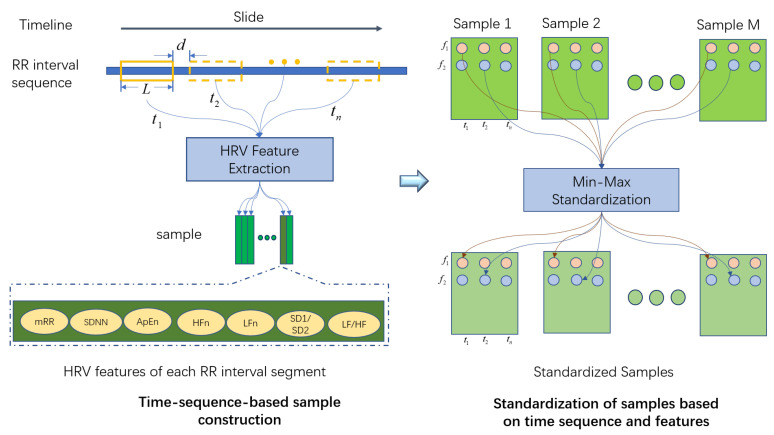
Sample construction and standardization.

In this section, sample construction and feature standardization processing of ECG signals are elaborated. The complete process is shown in Figure 6. The left of Figure 6 shows the sample construction process, and the right shows the sample standardization method. In the process of sample construction, we first intercept *N* RR interval segments of length *L* from the RR interval sequence with sliding step *d* according to the time series and calculate the HRV features corresponding to each segment. Finally, the HRV features of the RR interval segments are arranged in the sample matrix shown in Figure 6, where $t_{n}$ represents the moment corresponding to the *n*-th segment of the RR interval sequence. In this paper, we take *L* as 30 s, sliding step *d* as 2 s and *N* as 20. It should be noted that the physical meanings of the HRV features in the constructed samples are different, and there is a temporal relationship between the HRV feature sequences. Therefore, we use the min-max standardization method to standardize each feature at each moment in each sample based on the time series characteristics. The specific process is shown on the right of Figure 6. $f_{1}$ and $f_{2}$ represent different HRV features, and *M* represents the total number of samples. The final standardized samples can be obtained by processing the feature sequence composed of each feature at each moment in all samples. The calculation formula of the minimum and maximum standardization is shown in Equation (1):

(1) $${\hat{f}}_{i,j}=\frac{f_{i,j}-\min\left(F_{i,j}\right)}{\max\left(F_{i,j}\right)-\min\left(F_{i,j}\right)},$$

where $f_{i,j}$ is the HRV feature of the *i*-th row and the *j*-th column in the sample (its physical meaning is the *j*-th feature in the HRV feature sequence at the *i*-th moment), i=1,2,…,N, and j=1,2,…,7; $\max(F_{i,j})$ and $\min(F_{i,j})$ are the maximum and minimum values of HRV features in row *i* and column *j* in training samples; ${\hat{f}}_{i,j}$ is the standardized HRV feature.

### 4.3. GRU Model

**Figure 7:**
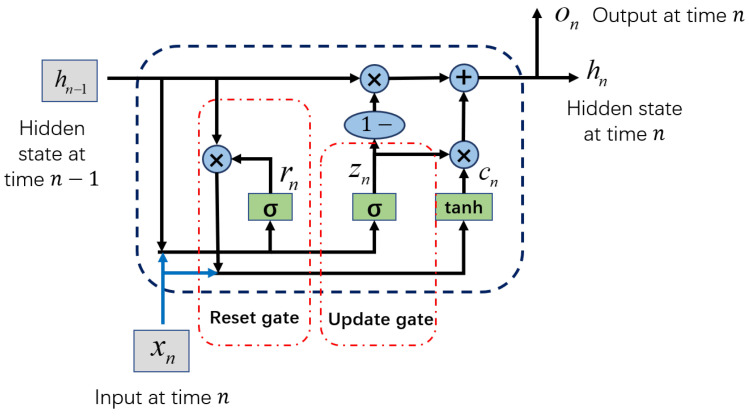
The GRU model structure.

GRU is a kind of recurrent neural network (RNN). GRU and Long Short-Term Memory (LSTM) are both proposed to solve the problem of gradient disappearance in the long-term dependence of learning time series in traditional RNN [35,36]. The performance of GRU and LSTM on many deep learning tasks is similar [37], but GRU has fewer parameters and less computation, so it has advantages in reducing the consumption of computing resources and the risk of overfitting. The structure of the GRU model is shown in Figure 7. Where the circles and ellipses with a blue background represent operators, the boxes with a green background represent functions and the boxes with a gray background represent inputs. There are two important gate functions in the GRU model: the update gate and reset gate. The function of the reset gate is to determine how much of the hidden state information of the previous moment will be added to the candidate state according to the current input and the hidden state of the previous moment, thereby generating the candidate state of the current moment. The function of the update gate is to determine which historical information in the hidden state at the previous moment can be forgotten and which information in the candidate state at the current moment can be added to the new hidden state, thereby generating the hidden state at the current moment. Equations (2) and (3) are the calculation formulas for the weights of the reset gate and the update gate, and the update formulas of the candidate state and the hidden state are shown in Equations (4) and (5) [37].

(2) $$r_{n}=\sigma \left(W_{ir}x_{n}+W_{hr}h_{n-1}\right),$$

(3) $$z_{n}=\sigma \left(W_{iz}x_{n}+W_{hz}h_{n-1}\right),$$

(4) $$c_{n}=\tanh\left(W_{ic}x_{n}+W_{hc}\left(r_{n}⊙h_{n-1}\right)\right),$$

(5) $$h_{n}=o_{n}=\left(1-z_{n}\right)⊙c_{n}+z_{n}⊙h_{n-1},$$

where $x_{n}$ is the input at the *n*-th moment, and $h_{n-1}$ is the hidden state at the n−1-th moment, $W_{ir}$ and $W_{hr}$ are the weight matrices of the reset gate input layer and the hidden state layer, $W_{iz}$ and $W_{hz}$ are the weight matrices of the update gate input layer and the hidden state layer, $W_{ic}$ and $W_{hc}$ are the input layer weight matrices and the hidden state layer weight matrices in the candidate state calculation, the bias matrices are all included by the weight matrices. $c_{n}$ is the candidate state at the *n*-th moment, and $h_{n}$ and $o_{n}$ are the hidden state and output at the *n*-th moment. Operator ⊙ is an element-wise multiplication. σ and tanh are activation functions, and their calculation formulas are $\sigma \left(x\right)=\frac{1}{1+e^{-x}}$ and $\tanh\left(x\right)=\frac{e^{x}-e^{-x}}{e^{x}+e^{-x}}$, respectively. They can improve the nonlinear capabilities of the model.

### 4.4. Psychological Stress Classification Model

**Figure 8:**
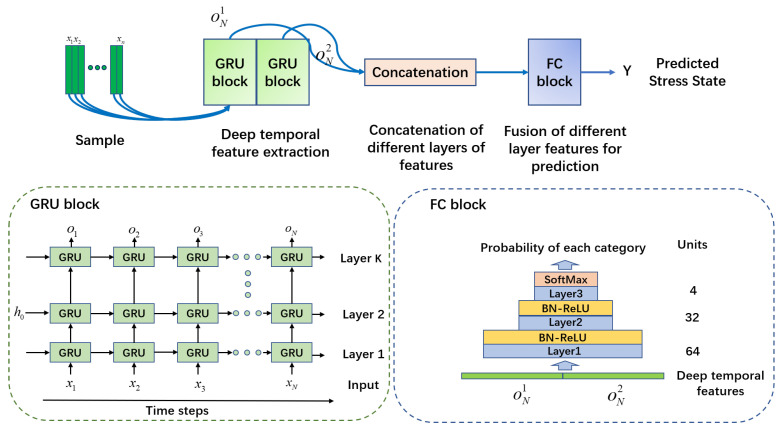
Psychological stress classification model.

The proposed psychological stress classification model consists of two sub-models, deep feature extraction and psychological stress classification, and its overall structure is shown in Figure 8. The deep feature extraction model is composed of two cascaded GRU blocks, and the output of each moment of the first block is input to the second block in the same order. The content in the green dotted box in Figure 8 shows the structure diagram of each GRU block expanded by time steps. Each GRU block is composed of *K* layers of the GRU network, and each layer of the GRU network contains *N* time step inputs, where $x_{n}$ and $o_{n}$ represent the input and output of the *n*-th moment, and the structure of each GRU model is shown in Figure 7. Considering that the output of the GRU network at the last moment contains the information of the entire sequence, the multi-level deep features composed of the outputs of the last moment of the two GRU blocks are used as the input of the psychological stress classification model. The psychological stress classification model consists of a multi-layer, fully connected network and a SoftMax classifier. As shown in the blue dotted box in Figure 8, there is a batch normalization layer between each layer of fully connected networks to standardize the distribution of neural network output, and ReLU is used as the activation function. The numbers in the FC block in Figure 8 are the number of neurons in each layer of the neural network.

The loss function used in the proposed method is shown in Equation (6), which consists of cross entropy loss and L2 regular loss. Cross entropy loss is used to measure the classification error of the model, and L2 loss is used to measure the complexity of the model to reduce the risk of overfitting. In training, the deep network model is optimized by minimizing the loss function.

(6) $$loss\left(y,\hat{y}\right)=-\frac{1}{M}\left(\sum_{i=1}^{M}\sum_{k=1}^{K}y_{i}^{\left(k\right)}log{\hat{y}}_{i}^{k}\right)+\gamma {∥W∥}_{2},$$

where *M* is the number of each batch sample in the training process, *K* is the total number of categories and *y* and $\hat{y}$ are the real labels and prediction probabilities of samples, respectively. γ is the hyperparameter of L2 loss, which controls the participation of L2 loss.

### 4.5. Evaluation Indicators

Since the number of samples under the four classes (psychological stress states) in this dataset is equal, the accuracy rate is used as an evaluation index to measure the performance of the methods. The accuracy of classification is the ratio of the number of correctly classified samples to the total number of samples, and its calculation formula is shown in Equation (7). When the number of samples in each category is balanced, the accuracy rate can objectively and intuitively show the classification accuracy of the algorithms. The higher the accuracy rate, the higher the classification performance of the algorithm.

(7) $$Accuracy=\frac{T_{1}+T_{2}+T_{3}+T_{4}}{M},$$

where *M* is the total number of samples, and $T_{1}$∼$T_{4}$ are the number of correctly predicted samples of category 1∼4, respectively.

## 5. Experiments and Results

### 5.1. Experimental Setting and Parameters

The proposed method is built and evaluated on the dataset mentioned in Section 3.3. In order to be more consistent with the actual applications, the cross-subject test method is used to verify the estimated performance of the method. In this paper, the training set consists of ECG data on 47 subjects, in which 8 subjects’ ECG data are randomly selected as the validation set, and the remaining 16 subjects’ ECG data are the test set. In data processing, each subject’s ECG signal under each stress state is broken into 20 ECG signal segments with a length of 30 s, and the interception interval is 2 s. These 20 signal segments correspond to the time steps of the GRU network inputs.

In the deep feature extraction model, each GRU block is formed by stacking 5 layers of the GRU network, each layer of the GRU network has 256 neurons, and the length of the input sequence is 20 time steps. The classification model is composed of three layers of fully connected networks stacked, and the number of neurons from the bottom layer to the top layer is 64, 32 and 4. During the training process, the L2 regularization coefficient γ is 0.002, the learning rate is 0.0002, the batch size is 52, the model is optimized by Adam and the number of epochs is about 80. The training ends when the accuracy of the training set and the validation set is high, and the accuracy of the validation set is stable. During validation and testing, the validation and test sets are standardized using the parameters in the training set. In order to evaluate the performance of the model more objectively, we conduct five independent repeated experiments and take the average of its accuracy as the final evaluation index.

### 5.2. Experimental Platform

The hardware configuration of the workstation for this experiment is Intel i7-11700F CPU with 16GB RAM, and NVIDIA 1060Ti GPU. The software platform is Python 3.7.11, Pytorch 1.10.0 and CUDA 11.3.

### 5.3. Results and Analysis

**Figure 9:**
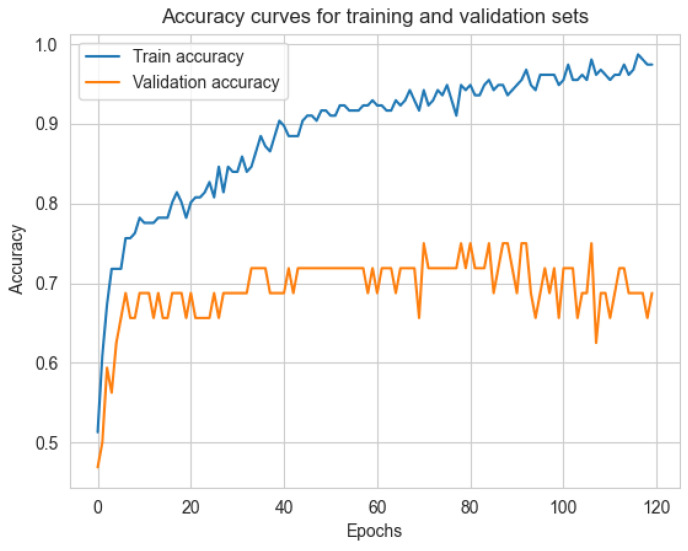
Accuracy curves of training set and validation set during training.

In this section, the psychological stress state estimation performance of the proposed method is presented and discussed from different perspectives. First, different numbers of HRV features are tried to train deep models to observe the contribution of different features to the estimation performance of the proposed method, and the results are shown in Table 2. When only mRR and ApEn features are used, the classification accuracy of the model is only 0.51. As more HRV features are added to the training of the model, its classification accuracy keeps rising and eventually reaching 0.73 when all HRV features are used. In addition, it can be seen that compared with the HRV features, for except mRR and ApEn, SDNN has a higher contribution to stress state classification performance. Then, Figure 9 shows the training set accuracy and validation set accuracy curves of the proposed algorithm during training. It can be seen that as the number of epochs increases, the estimated accuracy of the model in both the training set and the validation set keeps rising steadily. It should be noted that the gap between the accuracy of the training set and the accuracy of the validation set continues to increase as the training progresses, and the risk of overfitting the model will increase if the training continues. Therefore, the model with the iteration number of 80 epochs is selected for performance evaluation on the test set.

The classification performance of the proposed method and the comparison algorithms are presented in Table 3; the number in bold is the best result. Among them, comparison algorithms include the traditional machine learning algorithm KNN, ensemble learning method XGBoost [38], deep learning method MLP [23] and one-dimensional CNN (CNN-1D) network. KNN has been widely used in the study of psychological stress classification. XGBoost, as a state of art ensemble learning method, has been applied in many machine learning fields. In [23], a good psychological stress classification result is obtained by using the MLP method. At present, a one-dimensional CNN network is also widely used in the field of physiological signal processing and has achieved good results [39,40]. GRU-b1, GRU-b2 and GRU-b3 in Table 3 are the methods proposed in this paper, which represent the deep time series feature extraction model composed of one GRU block, two GRU blocks and three GRU blocks, respectively. It can be seen that the classification accuracy of psychological stress obtained by our methods is better than all comparison algorithms. When using two GRU blocks to extract deep features, the classification accuracy of psychological stress is the highest among all methods, reaching 0.78. We believe that this is because the long-short-term memory network may be able to capture longer-term dependencies between time series than other machine learning methods, thereby obtaining more global and robust characteristics of psychological stress. In addition, with the increase in GRU blocks, the classification performance of the model tends to increase first and then maintain or slightly decrease. This is because when there are fewer model parameters, the risk of model underfitting is high. When there are too many model parameters, it is easy for the model to fall into overfitting, which will reduce the classification performance of the model on the test set.

We also perform some exploration on the impact of other model hyperparameters on model performance; Table 4 and Table 5 show the estimated performance of the proposed algorithm under different model parameters. Table 4 shows the impact of GRU networks with different numbers of neurons on the model estimation performance, and Table 5 shows the impact of different numbers of GRU network layers on the model estimation performance. It can be seen that with the increasing number of neurons and network layers in the network, the classification accuracy of the model shows a trend of rising first and then declining under the influence of the risk of underfitting and overfitting. When the number of neurons is 256 and the number of network layers is 5, the classification accuracy of the model is the highest. Furthermore, it can be seen that the depth of the GRU network significantly affects the classification accuracy of the model. This is because, compared with the shallow network, the deep network can extract more essential features from HRV data. These features represent the common characteristics of physiological data of different subjects under the same stress state, and they can affect the generalization performance of the model.

**Figure 10:**
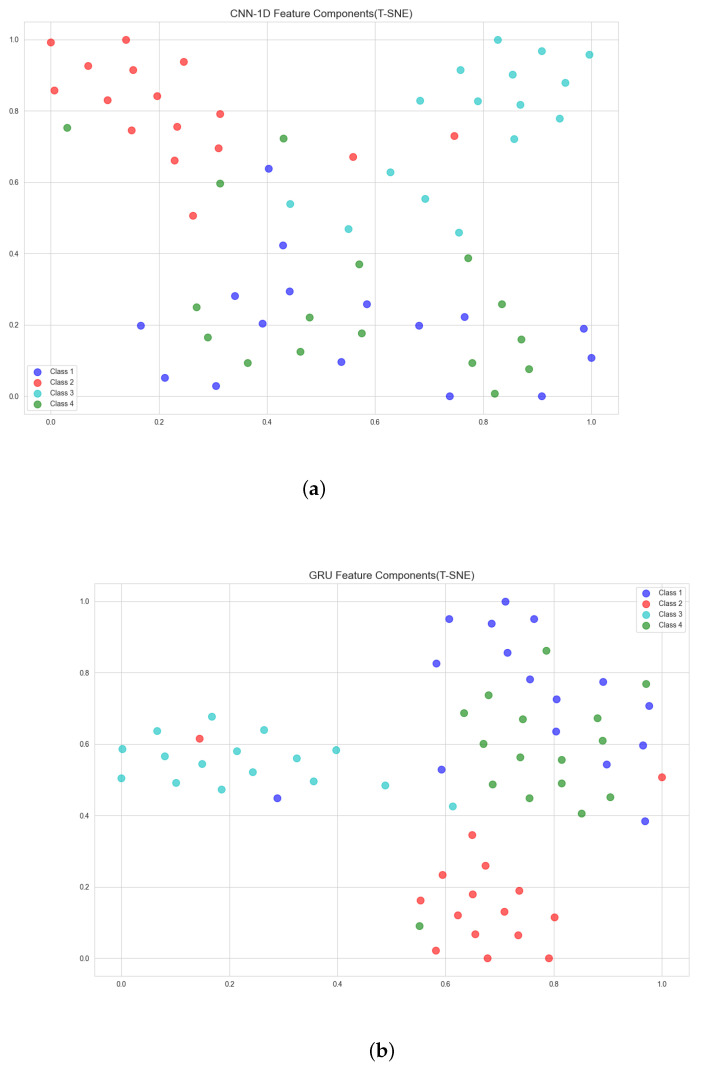
Feature extraction results of CNN-1D and the proposed method on subject HRV data. (**a**) The distribution of the features output by the CNN-1D model after T-SNE dimensionality reduction. (**b**) The distribution of the features output by the proposed method after dimensionality reduction by T-SNE.

In addition, we also explore the classification performance of the model in each class. Labels and classes 1, 2, 3 and 4 in Table 6 and Figure 10 represent four stress states, namely, resting, VR task, recovery and VR scene adaptation. Table 6 is the confusion matrix for the classification of the proposed method on the test set, and the data in the table is the proportion of the number of samples predicted to be in this class among all the samples in this class. It can be seen that the model has the lowest classification accuracy for class 1 (resting state), 38% of the samples are wrongly classified into class 4 (the state of adapting to the VR scene), and 13% of the samples in class 4 are also wrongly classified into class 1. This is because, in the process of adapting to the VR scene, the subjects only need to keep standing to observe and become familiar with the VR high-altitude scene, which induces less psychological stress so that the physiological parameters of the subjects at this time are very close to those at rest. This results in a lower classification accuracy of the model in class 1 compared to other classes. As shown in Figure 10, we also use the t-distributed Stochastic Neighbor-Embedding (T-SNE) [41] method to reduce the dimensionality and visualize the deep feature output by the CNN-1D and proposed method, respectively, where the dots with different colors represent the class to which the feature belongs. The position of each dot represents the distribution characteristics of the deep features extracted from a sample in the two-dimensional feature space. It can be seen from Figure 10a that the classification boundaries between the features of each class extracted by the CNN-1D model are relatively blurred, and the feature distribution of each category of samples is loose. The extracted features using the proposed deep model are shown in Figure 10b. It can be seen that compared with the CNN-1D model, the classification boundaries between the various categories of features extracted by our method are more obvious, and the distribution of sample features of each category is also more concentrated. Furthermore, we can see that the method proposed by us can also improve the separability between class 1 and class 4 samples to a certain extent, which indicates that our method extracts more essential psychological stress features from HRV data. Although our method has some improvement in feature extraction compared to the CNN-1D method, the classification boundaries of deep features of class 1 and class 4 are still blurred, which is consistent with the results in the confusion matrix. The identification of this weak-intensity stress is a difficult problem in the current research field of psychological stress estimation, and it will be also the focus of our future research.

## 6. Conclusions

This paper proposes a deep psychological stress classification method based on ECG signals. First, HRV feature samples containing the timing information of ECG signals are constructed. Deep GRU networks are then used to extract deep features from HRV feature samples that have more essential and general connections to psychological stress states. Finally, a multi-layer, fully connected network is used to fuse the deep and shallow features of the GRU network to predict the psychological stress state. The experimental results show that the proposed method is a robust psychological stress estimation scheme, and its estimation accuracy in this dataset is 0.78 better than other mainstream methods.

However, we noticed that the classification accuracy is not very high. In future work, we will try to further improve the accuracy of psychological stress classification from the following aspects. The first is that the amount of information input to the classification model can be increased by introducing other physiological signals besides ECG, such as EEG and EDA, or extracting more valuable features from ECG signals, thereby improving the performance of stress classification. Secondly, we can also consider reducing the differences in physiological signals between individuals to improve the classification accuracy of psychological stress. Specifically, domain adaptation methods in transfer learning have achieved good results in many image datasets with large distribution differences, and in recent years, this method has achieved high performance in EEG-based cross-subject emotion recognition accuracy [42,43]. Therefore, we will consider introducing a transfer learning method to further improve the classification accuracy of psychological stress states. Furthermore, high-level feature design and feature space applicable reduction to multidimensional wearable sensors, such as referable approaches for wearable-based HAR, are also worthy of further experimentation [14,44].

## Figures and Tables

**Table 1:** HRV features and their descriptions in the paper.

Feature	Description
mRR	The mean value of the RR interval (time between adjacent heartbeats) sequence.
SDNN	The standard deviation of RR intervals (time series of adjacent heartbeat intervals).
HFn	Normalized spectral energy of heart rate variability from 0.15 to 0.4 Hz.
LFn	Normalized spectral energy of heart rate variability from 0.04 to 0.15 Hz.
LF/HF	The ratio of low-frequency to high-frequency power for heart rate variability.
ApEn	The approximate entropy of the RR interval sequence, which is used to measure the complexity of the sequence.
SD1/SD2	In the point cloud data of the poincare plots drawn with the RR intervals, the variance of the distribution along the longer axis is SD2, and the variance of the distribution along the shorter axis is SD1. SD1/SD2 is the ratio of SD1 and SD2.

**Table 2:** Classification accuracy of the proposed method in stress state with different HRV features. In this sub-experiment, 28 subjects are randomly selected from the training set described in Section 5.1 to train the model, and 15 subjects are randomly selected from the remaining data for validation. The accuracy in the table is the average accuracy of three independent repeated experiments on the validation set. The number in bold represents the best result.

Features	Accuracy
mRR, ApEn	0.51
mRR, ApEn, SD1/SD2	0.56
mRR, ApEn, SD1/SD2, SDNN	0.68
mRR, ApEn, SD1/SD2, SDNN, HFn	0.67
mRR, ApEn, SD1/SD2, SDNN, HFn, LFn	0.71
mRR, ApEn, SD1/SD2, SDNN, HFn, LFn, LF/HF	**0.73**

**Table 3:** Psychological stress state classification accuracy of the proposed algorithm and comparison algorithms on the test set. The number in bold represents the best result.

Algorithms	KNN	XGBoost	MLP [23]	CNN-1D	GRU-b1	GRU-b2	GRU-b3
**Accuracy**	0.65	0.69	0.71	0.7	0.73	**0.78**	0.77

**Table 4:** The classification accuracy of the psychological stress of the GRU network of the proposed method under different numbers of neural units. The number in bold represents the best result.

**The number of GRU units**	64	128	256	512
**Accuracy **	0.75	0.75	**0.78**	0.73

**Table 5:** The classification accuracy of the psychological stress of the GRU block of the proposed method under different numbers of neural network layers. The number in bold represents the best result.

**The number of layers of GRU block**	1	3	5	7
**Accuracy **	0.67	0.7	**0.78**	0.76

**Table 6:** The classification confusion matrix of the proposed method for psychological stress states. The data in the table is the proportion of the number of samples predicted to be this label in all samples of this label.

Proportion		Predict Labels	1	2	3	4
		
True Labels						
**1**			0.56	0.06	0	0.38
**2**			0	0.88	0.06	0.06
**3**			0.06	0	0.94	0
**4**			0.13	0.06	0	0.81

## Data Availability

The data are not publicly available due to the relevant project regulations.
